# Supplementary material for: Performance of the ImmuView and BinaxNOW assays for the detection of urine and cerebrospinal fluid Streptococcus pneumoniae and Legionella pneumophila serogroup 1 antigen in patients with Legionnaires’ disease or pneumococcal pneumonia and meningitis
Source: PLoS One. 2020 Aug 31;15(8):e0238479. doi: 10.1371/journal.pone.0238479 (PMC7458278; doi:10.1371/journal.pone.0238479)
Supplement: S1 Table — (PDF) [file pone.0238479.s001.pdf]

S1 Table

Clinical Sensitivity and Specificity for *S. pneumoniae* Antigenuria by Study Site

Site	Sensitivity[a] (%)		Specificity[b] (%)	
	ImmuView	BinaxNOW	ImmuView	BinaxNOW
UPenn	29.0 ((7.5 to 64.8)/7[c]	57.0 (25.0 to 84.2)/7[d]	94.1 (89.0 to 97.0)/153[e]	96.7 (92.3 to 98.8)/153[f]
SSI	96.2 (86.1 to 99.6)/52[c]	100 (90.7 to 100)/47[d]	97.8 (94.3 to 99.3)/186[e]	97.3 (93.2 to 98.9)/184[f]

a, mean (95% CI)/total patients with pneumococcal bacteremia; b, mean (95% CI)/total patients without pneumococcal bacteremia, many of whom had bacteremia caused by other bacteria, or Legionnaires' disease; c, d, e, f, p= 0.0001, 0.001, 0.09 and 0.76 for c, d, e, f, respectively, by Fisher's exact test
