# Supplementary material for: Performance of the ImmuView and BinaxNOW assays for the detection of urine and cerebrospinal fluid Streptococcus pneumoniae and Legionella pneumophila serogroup 1 antigen in patients with Legionnaires’ disease or pneumococcal pneumonia and meningitis
Source: PLoS One. 2020 Aug 31;15(8):e0238479. doi: 10.1371/journal.pone.0238479 (PMC7458278; doi:10.1371/journal.pone.0238479)
Supplement: S2 Table — (PDF) [file pone.0238479.s002.pdf]

S2 Table
Agreement of *S. pneumoniae* Antigenuria Testing, UPenn

ImmuView	BinaxNOW	
	positive	negative
positive	6	5
negative	3	146

p = 0.72, McNemar test
