# Supplementary material for: Performance of the ImmuView and BinaxNOW assays for the detection of urine and cerebrospinal fluid Streptococcus pneumoniae and Legionella pneumophila serogroup 1 antigen in patients with Legionnaires’ disease or pneumococcal pneumonia and meningitis
Source: PLoS One. 2020 Aug 31;15(8):e0238479. doi: 10.1371/journal.pone.0238479 (PMC7458278; doi:10.1371/journal.pone.0238479)
Supplement: S3 Table — (PDF) [file pone.0238479.s003.pdf]

S3 Table

Agreement of *S. pneumoniae* Antigenuria Testing, SSI

| ImmuView | BinaxNOW | |
|---|---|---|
| | positive | negative |
| positive | 47 | 2 |
| negative | 5 | 177 |

p =0.45, McNemar test
