# Supplementary material for: Performance of the ImmuView and BinaxNOW assays for the detection of urine and cerebrospinal fluid Streptococcus pneumoniae and Legionella pneumophila serogroup 1 antigen in patients with Legionnaires’ disease or pneumococcal pneumonia and meningitis
Source: PLoS One. 2020 Aug 31;15(8):e0238479. doi: 10.1371/journal.pone.0238479 (PMC7458278; doi:10.1371/journal.pone.0238479)
Supplement: S4 Table — (PDF) [file pone.0238479.s004.pdf]

S4 Table

Agreement of L. pneumophila Antigenuria Testing Lp SG1 only, UPenn

ImmuView	BinaxNOW	
	positive	negative
positive	89	1
negative	3	54

p = 0.61, McNemar test
