# Supplementary material for: Performance of the ImmuView and BinaxNOW assays for the detection of urine and cerebrospinal fluid Streptococcus pneumoniae and Legionella pneumophila serogroup 1 antigen in patients with Legionnaires’ disease or pneumococcal pneumonia and meningitis
Source: PLoS One. 2020 Aug 31;15(8):e0238479. doi: 10.1371/journal.pone.0238479 (PMC7458278; doi:10.1371/journal.pone.0238479)
Supplement: S6 Table — (PDF) [file pone.0238479.s006.pdf]

S6 Table

Clinical Sensitivity and Specificity for *L. pneumophila* Antigenuria by Study Site

Site	Sensitivity[a] (%)		Specificity[b] (%)	
	ImmuView	BinaxNOW	ImmuView	BinaxNOW
UPenn[c]	91.8 (84.4 to 96.0)/98[d]	93.8 (89.6 to 97.3)/98[e]	100 (91.3 to 100)/49[f]	100 (91.3 to 100)/49[g]
SSI	80.0 (67.3 to 88.5)/55[d]	66.7 (53.2 to 77.7)/54[e]	99.5 (96.6 to 100)/178[f]	98.9 (95.7 to 99.9)/178[g]

[a] mean (95% CI)/total patients with Legionnaires' disease; [b] mean (95% CI)/total patients without Legionnaires' disease, many of whom had pneumococcal bacteremia, bacteremia caused by other bacteria or other respiratory pathogens isolated from sputum or lower respiratory specimens; [c] included in the UPenn Legionnaires' disease specimens, but omitted from the analysis were 7 urines from patients with Legionnaires' disease caused by *Legionella* spp other than *L. pneumophila* (*L. wadsworthii, L. bozemanae, L. longbeachae* SG1 (2)), or *L. pneumophila* serogroups other than serogroup 1 (SG 2, SG4 (2)); none were detected by either assay. In addition, also excluded from analysis are seven specimens from patients suspected of Legionnaires' disease because of a Legionnaires' disease outbreak or sporadic cases, and who had originally equivocal urine antigen assays and negative cultures. If these non- L. pneumophila serogroup 1 infections and suspect cases are included, UPenn test specificity is unchanged but both BinaxNOW and ImmuView sensitivity changed to 85.0 (76.7 to 90.3)/111; [d,e,f,g] p= 0.001, <0.0001, 1 and 1 by Fisher exact test, for d, e, f and g, respectively.
