# Supplementary material for: Performance of the ImmuView and BinaxNOW assays for the detection of urine and cerebrospinal fluid Streptococcus pneumoniae and Legionella pneumophila serogroup 1 antigen in patients with Legionnaires’ disease or pneumococcal pneumonia and meningitis
Source: PLoS One. 2020 Aug 31;15(8):e0238479. doi: 10.1371/journal.pone.0238479 (PMC7458278; doi:10.1371/journal.pone.0238479)
Supplement: S7 Table — (PDF) [file pone.0238479.s007.pdf]

S7 Table

Correlation of BinaxNOW and ImmuView *L. pneumophila* Results and Monoclonal Group for Concordant and Discordant Results, SSI

BinaxNOW-ImmuView Results Discordant?	Monoclonal Group	
	Pontiac	Not-Pontiac
yes	5	2
no	18	23

p = 0.2 by Fisher's exact test
