# Supplementary material for: Performance of the ImmuView and BinaxNOW assays for the detection of urine and cerebrospinal fluid Streptococcus pneumoniae and Legionella pneumophila serogroup 1 antigen in patients with Legionnaires’ disease or pneumococcal pneumonia and meningitis
Source: PLoS One. 2020 Aug 31;15(8):e0238479. doi: 10.1371/journal.pone.0238479 (PMC7458278; doi:10.1371/journal.pone.0238479)
Supplement: S8 Table — (PDF) [file pone.0238479.s008.pdf]

S8 Table

Correlation of BinaxNOW *L. pneumophila* Falsely-negative Tests with Monoclonal Group, SSI Site

Urine Specimen #	Patient Sputum Culture *L. pneumophila* Monoclonal Group	ImmuView Result	BinaxNOW Result	Results after Boiling and Retesting	
				ImmuView	BinaxNOW
17	Pontiac	Positive	Negative	Negative	not performed
115	not Pontiac	Positive	Negative	Negative	not performed
169	Pontiac	Positive	Negative	Positive	not performed
189	Pontiac	Positive	Negative	Positive	not performed
194	Pontiac	Positive	Negative	Positive	not performed
204	Pontiac	Positive	Negative	Positive	Negative
222	not Pontiac	Positive	Negative	Negative	Negative
