# Supplementary material for: Performance of the ImmuView and BinaxNOW assays for the detection of urine and cerebrospinal fluid Streptococcus pneumoniae and Legionella pneumophila serogroup 1 antigen in patients with Legionnaires’ disease or pneumococcal pneumonia and meningitis
Source: PLoS One. 2020 Aug 31;15(8):e0238479. doi: 10.1371/journal.pone.0238479 (PMC7458278; doi:10.1371/journal.pone.0238479)
Supplement: S9 Table — (PDF) [file pone.0238479.s009.pdf]

S9 Table

Agreement for UPenn Site Only of BinaxNOW and ImmuView *L. pneumophila* Assays Including Urines from Patients with *Legionella* infections other than non-*L. pneumophila* serogroup 1, Culture-negative Outbreak Suspects and Culture-negative Sporadic Cases with Initial Borderline-positive Urine Antigen Tests[a]

	BinaxNOW	
ImmuView	positive	negative
positive	91	3
negative	3	63

p=1.0, McNemar test

[a] This includes, in addition to the results shown in Table 5, seven patients with culture-positive Legionnaires' disease caused by *L. wadsworthii*, *L. bozemanae*, *L. longbeachae* serogroup 1 (2 patients), *L. pneumophila* serogroup 2 and *L. pneumophila* serogroup 4 (2 patients), all of which were negative in both assays; three patients who were suspects in two different Legionnaires' disease outbreaks caused by *L. pneumophila* serogroup 1, but were originally culture negative and had borderline-positive urine antigen tests at the time of original collection; and three suspected sporadic cases of Legionnaires' disease who had borderline-positive urine antigen tests and negative cultures at the time of collection.
