# Supplementary material for: Performance of the ImmuView and BinaxNOW assays for the detection of urine and cerebrospinal fluid Streptococcus pneumoniae and Legionella pneumophila serogroup 1 antigen in patients with Legionnaires’ disease or pneumococcal pneumonia and meningitis
Source: PLoS One. 2020 Aug 31;15(8):e0238479. doi: 10.1371/journal.pone.0238479 (PMC7458278; doi:10.1371/journal.pone.0238479)
Supplement: S10 Table — SSI and UPenn sites combined. (PDF) [file pone.0238479.s010.pdf]

S10 Table

Agreement of BinaxNOW and ImmuView L. pneumophila Assays Including Urines from UPenn Patients with *Legionella* infections other than non-*L. pneumophila* serogroup 1, Culture-negative Outbreak Suspects and Culture-negative Sporadic Cases with Initial Borderline-positive Urine Antigen Tests[a]. SSI and UPenn Sites Combined.

	BinaxNOW	
ImmuView	positive	negative
positive	127	11
negative	5	249

p=0.21, McNemar test

[a] This includes, in addition to the results shown in Table 5, seven patients with culture-positive Legionnaires' disease caused by *L. wadsworthii*, *L. bozemanae*, *L. longbeachae* serogroup 1 (2 patients), *L. pneumophila* serogroup 2 and *L. pneumophila* serogroup 4 (2 patients), all of which were negative in both assays; three patients who were suspects in two different Legionnaires' disease outbreaks caused by *L. pneumophila* serogroup 1, but were originally culture negative and had borderline-positive urine antigen tests at the time of original collection; and three suspected sporadic cases of Legionnaires' disease who had borderline-positive urine antigen tests and negative cultures at the time of collection.
