# Supplementary material for: Performance of the ImmuView and BinaxNOW assays for the detection of urine and cerebrospinal fluid Streptococcus pneumoniae and Legionella pneumophila serogroup 1 antigen in patients with Legionnaires’ disease or pneumococcal pneumonia and meningitis
Source: PLoS One. 2020 Aug 31;15(8):e0238479. doi: 10.1371/journal.pone.0238479 (PMC7458278; doi:10.1371/journal.pone.0238479)
Supplement: S11 Table — SSI and UPenn combined data. (PDF) [file pone.0238479.s011.pdf]

S11 Table

BinaxNOW and ImmuView *L. pneumophila* Clinical Sensitivity and Specificity When Including Specimens from Patients with *Legionella* infections other than non-*L. pneumophila* serogroup 1, Culture-negative Outbreak Suspects and Culture-negative Sporadic Cases with Initial Borderline-positive Urine Antigen Tests[a]. SSI and UPenn Combined Data.

Assay	Sensitivity[b]	Specificity[c]
ImmuView	83.1 (76.6 to 88.0)/166	99.6 (97.3 to 100)/232
BinaxNOW	78.8 (71.9 to 84.3)/165	99.1 (96.6 to 99.9)/227

[a] This includes, in addition to the results shown in Table 5, seven patients with culture-positive Legionnaires' disease caused by *L. wadsworthii*, *L. bozemanae*, *L. longbeachae* serogroup 1 (2 patients), *L. pneumophila* serogroup 2 and *L. pneumophila* serogroup 4 (2 patients), all of which were negative in both assays; three patients who were suspects in two different Legionnaires' disease outbreaks caused by *L. pneumophila* serogroup 1, but were originally culture negative and had borderline-positive urine antigen tests at the time of original collection; and three suspected sporadic cases of Legionnaires' disease who had borderline-positive urine antigen tests and negative cultures at the time of collection; [b] mean (95% CI)/total patients with Legionnaires' disease; [c] mean (95% CI)/total patients without Legionnaires' disease, many of whom had pneumococcal bacteremia, bacteremia caused by other bacteria or other respiratory pathogens isolated from sputum or lower respiratory specimens
