# Supplementary material for: Performance of the ImmuView and BinaxNOW assays for the detection of urine and cerebrospinal fluid Streptococcus pneumoniae and Legionella pneumophila serogroup 1 antigen in patients with Legionnaires’ disease or pneumococcal pneumonia and meningitis
Source: PLoS One. 2020 Aug 31;15(8):e0238479. doi: 10.1371/journal.pone.0238479 (PMC7458278; doi:10.1371/journal.pone.0238479)
Supplement: S13 Table — (PDF) [file pone.0238479.s013.pdf]

S13 Table

Positive and Negative Agreement for CSF Specimens

Assay	Target	Positive Agreement[a] (%)	Negative Agreement[b] (%)
ImmuView	*S. pneumoniae*	96.7 (81.6 to 100)/30	97.7 (95.1 to 98.9)/300
BinaxNOW		92.3 (64.2 to 100)/13	98.9 (95.9 to 99.9)/187

a, mean (95% CI)/total positive specimens (meningitis and contrived meningitis); b, mean (95% CI)/total negative CSF specimens (contrived).
