# Supplementary material for: Performance of the ImmuView and BinaxNOW assays for the detection of urine and cerebrospinal fluid Streptococcus pneumoniae and Legionella pneumophila serogroup 1 antigen in patients with Legionnaires’ disease or pneumococcal pneumonia and meningitis
Source: PLoS One. 2020 Aug 31;15(8):e0238479. doi: 10.1371/journal.pone.0238479 (PMC7458278; doi:10.1371/journal.pone.0238479)
Supplement: S14 Table — (PDF) [file pone.0238479.s014.pdf]

S14 Table

S. pneumoniae Pediatric Urines Agreements

	BinaxNOW	
ImmuView	positive	negative
positive	3	0
negative	2	51

p=0.48, McNemar test
