# Supplementary material for: Performance of the ImmuView and BinaxNOW assays for the detection of urine and cerebrospinal fluid Streptococcus pneumoniae and Legionella pneumophila serogroup 1 antigen in patients with Legionnaires’ disease or pneumococcal pneumonia and meningitis
Source: PLoS One. 2020 Aug 31;15(8):e0238479. doi: 10.1371/journal.pone.0238479 (PMC7458278; doi:10.1371/journal.pone.0238479)
Supplement: S16 Table — (PDF) [file pone.0238479.s016.pdf]

S16 Table
Clinical Specificity for Pediatric Urines

Assay	Target	Specificity[a] (%)
ImmuView	*S. pneumoniae*	94.6 (84.7 to 98.7)/56
BinaxNOW		91.1 (80.2 to 96.5)/56
ImmuView	*L pneumophila*	100 (92.1 to 100)/56
BinaxNOW		100 (92.0 to 100)/55

a, mean (95% CI)/total patients without pneumococcal infection
