# Supplementary material for: Performance of the ImmuView and BinaxNOW assays for the detection of urine and cerebrospinal fluid Streptococcus pneumoniae and Legionella pneumophila serogroup 1 antigen in patients with Legionnaires’ disease or pneumococcal pneumonia and meningitis
Source: PLoS One. 2020 Aug 31;15(8):e0238479. doi: 10.1371/journal.pone.0238479 (PMC7458278; doi:10.1371/journal.pone.0238479)
Supplement: S17 Table — (PDF) [file pone.0238479.s017.pdf]

S17 Table

Effects of Retesting Urine after Boiling for ImmuView-BinaxNOW Discordant Result Specimens for Upenn and SSI Sites[a]

Result Change After Boiling[b]	*S. pneumoniae*		*L. pneumophila*	
	ImmuView	BinaxNOW	ImmuView	BinaxNOW
FN to FN	2	0	1	4
FN to TP	1	1	1	1
TP to FN	0	0	3	0
TP to TP	1	1	7	2
TN to TN	3	3	0	0
TN to FP	3	0	0	0
FP to FP	2	1	0	0
FP to TN	4	0	1	0

[a]These are summary results and not paired comparisons. The UPenn site tested five *L pneumophila* and six *S. pneumonia*e ImmuView-BinaxNOW discordant specimens with both BinaxNOW and ImmuView tests. The SSI site tested eight *L. pneumophil*a ImmuView-BinaxNOW discordant specimens using only the ImmuView test for seven urines because of limited urine volumes and both BinaxNOW and Immuview for two specimens, and eight *S. pneumoniae* discordant specimens using only the Immuview test because of limited urine volumes; [b] FN, false-negative; TP, true-positive; TN, true-negative; FP, false-positive; FN to FN means false-negative before and after boiling, FN to TP means false-negative before boiling and true-positive after boiling, etc.
