# Supplementary material for: Performance of the ImmuView and BinaxNOW assays for the detection of urine and cerebrospinal fluid Streptococcus pneumoniae and Legionella pneumophila serogroup 1 antigen in patients with Legionnaires’ disease or pneumococcal pneumonia and meningitis
Source: PLoS One. 2020 Aug 31;15(8):e0238479. doi: 10.1371/journal.pone.0238479 (PMC7458278; doi:10.1371/journal.pone.0238479)
Supplement: Attachment — Submitted filename: response to reviewers.docx [file pone.0238479.s018.docx]

20 July 2020

**Re: PONE-D-20-15595
Performance of the ImmuView and BinaxNOW assays for the detection of urinary and cerebrospinal fluid Streptococcus pneumoniae and Legionella pneumophila serogroup 1 antigen in Legionnaires’ disease or pneumococcal pneumonia and meningitis
PLOS ONE**

Dear Editor:

Please find below our responses to the reviewers shown in bolded font. A copy of the manuscript showing the changes made, as well as an unmarked final copy of the manuscript are enclosed. We thank the reviewers for fair and comprehensive reviews, and believe that we have answered all their comments and queries satisfactorily.

Sincerely yours,

Paul H. Edelstein, M.D.

Reviewers' comments:

Reviewer's Responses to Questions

**Comments to the Author**

1. Is the manuscript technically sound, and do the data support the conclusions?

The manuscript must describe a technically sound piece of scientific research with data that supports the conclusions. Experiments must have been conducted rigorously, with appropriate controls, replication, and sample sizes. The conclusions must be drawn appropriately based on the data presented.

Reviewer #1: Yes

Reviewer #2: Partly

2. Has the statistical analysis been performed appropriately and rigorously?

Reviewer #1: I Don't Know

Reviewer #2: Yes

3. Have the authors made all data underlying the findings in their manuscript fully available?

The [PLOS Data policy](http://www.plosone.org/static/policies.action#sharing) requires authors to make all data underlying the findings described in their manuscript fully available without restriction, with rare exception (please refer to the Data Availability Statement in the manuscript PDF file). The data should be provided as part of the manuscript or its supporting information, or deposited to a public repository. For example, in addition to summary statistics, the data points behind means, medians and variance measures should be available. If there are restrictions on publicly sharing data—e.g. participant privacy or use of data from a third party—those must be specified.

Reviewer #1: Yes

Reviewer #2: Yes

4. Is the manuscript presented in an intelligible fashion and written in standard English?

PLOS ONE does not copyedit accepted manuscripts, so the language in submitted articles must be clear, correct, and unambiguous. Any typographical or grammatical errors should be corrected at revision, so please note any specific errors here.

Reviewer #1: Yes

Reviewer #2: Yes

5. Review Comments to the Author

Please use the space provided to explain your answers to the questions above. You may also include additional comments for the author, including concerns about dual publication, research ethics, or publication ethics. (Please upload your review as an attachment if it exceeds 20,000 characters)

Reviewer #1: Overall a good paper describing the results of a multisite study comparing the ImmunView and BinaxNOW urine antigen and CSF tests for Streptococcus pneumoniae and Legionella pneumophila. I would like to have seen more samples from patients with bacteraemic pneumococcal pneumonia tested at the UPenn site as I feel that the lack of these samples makes site comparisons for this particular test group difficult. I also have some minor points for you to consider.

1. Line 47- I don’t think it is necessary to describe the disease as “pneumonia caused by either pneumococcal pneumonia”. I would suggest something like “pneumococci” instead of “pneumococcal pneumonia” or alternatively remove the "caused by " and just list pneumococcal pneumonia.

**Done, Now reads “These assays were carried out at one diagnostic laboratory in the USA and one in Denmark using a large number of archived frozen urine specimens specific to each laboratory, from patients with pneumococcal pneumonia or Legionnaires' disease, or neither disease.”**

2. Line 59- Please could you list the criteria that was used to define the children with lower respiratory infection not due to pneumococcal pneumonia. As cases of co-infections with pneumococci and other bacteria or viruses have been reported, was this considered when selecting these samples?

**This is now detailed in the methods section:** **“Urines from 56 children with lower respiratory infection not due to pneumococcal pneumonia and known *S. pneumoniae* nasopharyngeal colonization status, were tested at SSI only. The criteria for pneumonia were clinical signs and symptoms of pneumonia. All children had respiratory specimens submitted for culture; none grew *S. pneumoniae*, while 12 grew *Staphylococcus aureus*, three *Branhamella catarrhalis*, one *Acinetobacter* spp., and the rest normal upper respiratory flora. Seven of the children had positive prior testing on their urines for pneumococcal antigen using the Binax NOW *S. pneumoniae* antigen card, with the remainder being negative. All but four of the children had nasopharyngeal cultures obtained to determine *S. pneumoniae* colonization on the same day that urines were collected for antigen testing, with 13 children found to be colonized. Culture and prior pneumococcal antigen testing results were not used to select these specimens, with the exception that no child was included if the respiratory tract culture grew *S. pneumoniae*. The age of the children ranged from <1 to 19 years, with a median of 5.5 years and mean of 6.6 years.”**

3. Line 172-174- The footnote for Table 1, specifically references e and f, are used to describe the total "urines" tested. However, the data in the first column of the table are supposed to represent CSF samples. Please can you clarify.

**Fixed. Footnotes now read “ ^e^ total positive/total negative patient urines or CSF specimens tested using ImmuView assay; ^f^ total positive/total negative patient urines or CSF specimens tested using BinaxNOW assays”**

4. Line 265 - Please can you clarify as to what is meant by the ImmunView L. pneumophila test. I am assuming that it means that some of the samples reported a result for L. pneumophila when tested with the ImmunView test.

**Fixed, now reads “The ImmunView *L. pneumophila* component of the dual target test strip,”**

5. Line 283-286 -Please can you explain why priority was given to the ImmunView test over the BinaxNow when conducting repeat testing with insufficient sample volume.

**Clarified by addition of the following: “Priority was given to testing the ImmuView test in the case of insufficient volume for both tests because less was known about the effect of specimen boiling on ImmuView results than is known about this effect on Binax assays.”**

6. Line 381-383 - Have you considered the sensitivity of the assays in regard to the different pneumococcal serotypes?

**No change made in the manuscript as we did not analyze test sensitivity in regard to pneumococcal serotype**

7. Table 4 - Could this table also include the figures for the number of samples reported positive and negative out of the total tested that was used to calculate the sensitivity and specificity?

**Done. The footnotes were also changed to explain the new notation**

Reviewer #2: In the present study, Edelstein et al compared the performances of ImmuView S. pneumoniae and L. pneumophila urinary antigen tests with that of the BinaxNOW S. pneumoniae and L. pneumophila assays using frozen samples and performing the testing at three laboratories. The study sponsor SSI Diagnostica wrote the test protocols and financed the study, but the report was written independently of them. The report adds information regarding the ImmuView test performance in comparison with the BinaxNOW assays on clinical samples.

My major concern is that samples are referred to as collected from pneumococcal pneumonia and Legionnaires’ disease patients but data are lacking on collection procedure and patient characteristics. When and where did the authors collect samples, and when were they analyzed? If the 55 Legionella serogroup 1 samples tested at SSI are the same 55 samples analyzed by Jorgensen et al (2015) previously this should be presented. Patient characteristics from those with suspected Legionnaires’ disease would help to interpret discordant results, especially since more than half of Legionnaires’ disease cases from the UPenn site are defined only by previous positive antigen tests (see below). It would be suitable to refrain from using the term “clinical” for defining test performance to avoid misunderstandings concerning the trial design.

**-Regarding the 55 SSI specimens, these were not the same specimens tested by Jorgensen et al. This is now stated in the methods section “The 55 positive urine specimens from patients with Legionnaires' disease tested by SSI were novel and not previously used in a prior published evaluation of the ImmuView or any other test[5].”**

**-****Regarding the clinical findings of the Legionnaires’ disease patients and control patients, all of the patients with Legionnaires' disease, regardless of culture positivity, had clinical findings diagnostic of acute pneumonia, including new pulmonary infiltrates and other signs and symptoms of acute pneumonia. All of the patients with bacteremic pneumococcal pneumonia also had clinical findings diagnostic of acute pneumonia, including new pulmonary infiltrates. The negative control patients for Legionnaires' disease included those with bacteremic pneumococcal pneumonia and patients who were felt to have pneumonia by their clinicians, who had requested microbiologic testing for causes of pneumonia. We do not know the clinical findings for the non-pneumococcal pneumonia negative-control patients. Further specifying the clinical findings for the Legionnaires' disease patients or pneumococcal pneumonia patients is not relevant here because the clinical findings of Legionnaires’ disease and pneumococcal pneumonia are not specific enough to be used to classify the etiologic certainty of these diseases based on clinical findings alone. Thus, the use of the terms “clinical sensitivity and specificity” are accurate. This is now stated in the methods section as “All patients with a diagnosis of Legionnaires' disease or bacteremic pneumococcal pneumonia had clinical findings consistent with acute pneumonia.** **All patients with previously negative tests for Legionnaires' disease or pneumococcal pneumonia were suspected of having bacterial pneumonia by their ordering clinicians.”**

**-We don’t understand what the reviewer means by specifying how a urine specimen was collected – if the reviewer means if the urines were spontaneously voided or collected by catheterization we do not have that information, and for the purposes of this analysis the method of collection is irrelevant. The methods section states that these cultures were stored frozen, sometimes for decades, before testing, at each site performing the test. Specimens were frozen at ≤-20C after being stored at 5C for up to seven days.**

Another concern is the lack of criteria for pneumonia diagnosis. Usually, a majority of patients with pneumococcal bacteremia have airway infections but, sometimes, may have meningitis, otitis or other foci only. If not described, maybe the authors should refer to patients with bacteremia and not bacteremic pneumonia. Furthermore, are these urine samples collected consecutively during certain time periods, or are they selected for some reason due to special features, e.g. if a positive pneumococcal antigen test was positive in the clinical routine along with positive blood culture? Could the selection of samples explain the high sensitivity yields for pneumococcal antigen testing for both tests in the study, and for site specific differences?

**-We have clarified that only one specimen was acquired from each patient and were not the result of consecutive collection in the methods section. “****The urine specimens tested were not the result of consecutive sampling during infection, were not selectively collected because of specific clinical findings, and were collected during the initial presentation of the pneumonia with only one specimen collected per patient.”**

Line 115: How was serogroup 1 identified?
**By immunologic means using serogroup 1-specific antibody. No changes were made in the text in response to this comment.**
Line 118: How was pneumonia defined?

**New pulmonary infiltrate and clinical findings consistent with pneumonia. This is now stated in the methods section.**

Line 155: Bacteremic pneumococcal pneumonia. Since the definition of Legionnaires’ disease and pneumococcal pneumonia is not clear, and based on positive microbiology results only, it should be referred to possible diagnoses.

**Please see the above responses to this query, repeated here for clarity.** **- Regarding the clinical findings of the Legionnaires’ disease patients and control patients, all of the patients with Legionnaires' disease, regardless of culture positivity, had clinical findings diagnostic of acute pneumonia, including new pulmonary infiltrates and other signs and symptoms of acute pneumonia. All of the patients with bacteremic pneumococcal pneumonia also had clinical findings diagnostic of acute pneumonia, including new pulmonary infiltrates. The negative control patients for Legionnaires' disease included those with bacteremic pneumococcal pneumonia and patients who were felt to have pneumonia by their clinicians, who had requested microbiologic testing for causes of pneumonia. We do not know the clinical findings for the non-pneumococcal pneumonia negative-control patients. Further specifying the clinical findings for the Legionnaires' disease patients or pneumococcal pneumonia patients is not relevant here because the clinical findings of Legionnaires’ disease and pneumococcal pneumonia are not specific enough to be used to classify the etiologic certainty of these diseases based on clinical findings alone. Thus, the use of the terms “clinical sensitivity and specificity” are accurate. This is now stated in the methods section as “All patients with a diagnosis of Legionnaires' disease or bacteremic pneumococcal pneumonia had clinical findings consistent with acute pneumonia.** **All patients with previously negative tests for Legionnaires' disease or pneumococcal pneumonia were suspected of having bacterial pneumonia by their ordering clinicians.”**

Line 157: The sentence refers to Table 1 only.
**Correction made, thank you.**
Line 178: Supportive laboratory findings for patients with suspected Legionnaires disease?
**We have no records of the “supportive” laboratory findings for these patients. There are no specific or sensitive “supportive” laboratory findings for Legionnaires’ disease other than microbiologic or molecular detection of the organism, or seroconversion to the bacterium. There is no relevance of listing such findings because of their limited sensitivity and specificity. No changes were made in the manuscript in response to this query.**

Line 199: Not clinical performance, since the definitions of diagnoses have not yet been clarified.
**See responses to this above. No changes were made in the manuscript based on this query.**
Line 210: Not clinical specificity, since the definitions of diagnoses have not yet been clarified.
**See responses to this above. No changes were made in the manuscript based on this query.**
Line 261: Not clinical sensitivity, half the samples are spiked.
**We agree with the reviewer that since not all CSF specimens were from patients with meningitis that the use of clinical sensitivity and specificity is not accurate. These terms were changed to positive and negative agreement in the text and Table S13.**
Line 269: Patient characteristics? Age? Previous urinary antigen test performed? Was urine sample and nasopharynx sample collected simultaneously, or colonization “at any time”?
**The ages of the children are now given in the methods section and other requested information is now given in the methods section. “Urines from 56 children with lower respiratory infection not due to pneumococcal pneumonia and known *S. pneumoniae* nasopharyngeal colonization status, were tested at SSI only. The criteria for pneumonia were clinical signs and symptoms of pneumonia. All children had respiratory specimens submitted for culture; none grew *S. pneumoniae*, while 12 grew *Staphylococcus aureus*, three *Branhamella catarrhalis*, one *Acinetobacter* spp., and the rest normal upper respiratory flora. Seven of the children had positive prior testing on their urines for pneumococcal antigen using the Binax NOW *S. pneumoniae* antigen card, with the remainder being negative. All but four of the children had nasopharyngeal cultures obtained to determine *S. pneumoniae* colonization on the same day that urines were collected for antigen testing, with 13 children found to be colonized. Culture and prior pneumococcal antigen testing results were not used to select these specimens, with the exception that no child was included if the respiratory tract culture grew *S. pneumoniae*. The age of the children ranged from <1 to 19 years, with a median of 5.5 years and mean of 6.6 years.”**

Line 340: Is it 54% (60/111)? Please refer to Table 2 If appropriate.
**Corrected, thank you**

Line 352: Due to absent test concordance…? Words missing.
**Language clarified**

Line 404: Little or no information of samples tested for specificity is presented in the study. Thus, if the samples are from healthy individuals, the results only reflects specificity on such a healthy population without respiratory symptoms. It would be of interest to study the test performances on patients with non-Legionella non-pneumococcal lower airway infections or bacteremia.
**With the exception of the CSF specimens, all other specimens were obtained from patients with clinical findings consistent with pneumonia. This is now clarified in the methods section. See comments above for details.**

Line 408: Here, it is stated that the use of the term clinical sensitivity is not appropriate in the study, I agree.
**The manuscript does not say that use of the term clinical sensitivity is not appropriate. Rather it says that because the patient specimens were biased to include those known to be antigen positive that concordance between the two test methods is the best measure of test performance and that our stated clinical performance data may be misleading because of this bias.**

Lines 388-391: If the selection of urine samples are based on positive Legionella urinary antigen tests only, with no clinical data on pneumonia, samples may be true-negative from the beginning, but false-positive before boiling. This is a major issue of the study, see above. Besides the discrepant specimens, could there be more positive tests that would have turned negative after boiling?

**We know that all studied patients with Legionnaires’ disease has clinically defined pneumonia, as is now clarified in the methods section. The urine boiling data are included in the results, but are not included in any of the calculations of test performance.**
