# Supplementary material for: Performance of the ImmuView and BinaxNOW assays for the detection of urine and cerebrospinal fluid Streptococcus pneumoniae and Legionella pneumophila serogroup 1 antigen in patients with Legionnaires’ disease or pneumococcal pneumonia and meningitis
Source: PLoS One. 2020 Aug 31;15(8):e0238479. doi: 10.1371/journal.pone.0238479 (PMC7458278; doi:10.1371/journal.pone.0238479)
Supplement: Attachment — Submitted filename: response to reviewer.docx [file pone.0238479.s019.docx]

13 August 2020

**Re: PONE-D-20-15595
Performance of the ImmuView and BinaxNOW assays for the detection of urinary and cerebrospinal fluid Streptococcus pneumoniae and Legionella pneumophila serogroup 1 antigen in Legionnaires’ disease or pneumococcal pneumonia and meningitis
PLOS ONE**

Dear Editor:

Please find below our responses to reviewer #1, from the 13 August 2020 review of the revised manuscript shown in bolded font. A copy of the manuscript showing the changes made, as well as an unmarked final copy of the manuscript are enclosed. We thank the reviewer for a fair and comprehensive reviews, and believe that we have answered all of the reviewer’s comments and queries satisfactorily.

Sincerely yours,

Paul H. Edelstein, M.D.

Reviewers' comments:

Reviewer's Responses to Questions

**Comments to the Author**

1. If the authors have adequately addressed your comments raised in a previous round of review and you feel that this manuscript is now acceptable for publication, you may indicate that here to bypass the “Comments to the Author” section, enter your conflict of interest statement in the “Confidential to Editor” section, and submit your "Accept" recommendation.

Reviewer #1: All comments have been addressed

2. Is the manuscript technically sound, and do the data support the conclusions?

The manuscript must describe a technically sound piece of scientific research with data that supports the conclusions. Experiments must have been conducted rigorously, with appropriate controls, replication, and sample sizes. The conclusions must be drawn appropriately based on the data presented.

Reviewer #1: Yes

3. Has the statistical analysis been performed appropriately and rigorously?

Reviewer #1: I Don't Know

4. Have the authors made all data underlying the findings in their manuscript fully available?

The [PLOS Data policy](http://www.plosone.org/static/policies.action#sharing) requires authors to make all data underlying the findings described in their manuscript fully available without restriction, with rare exception (please refer to the Data Availability Statement in the manuscript PDF file). The data should be provided as part of the manuscript or its supporting information, or deposited to a public repository. For example, in addition to summary statistics, the data points behind means, medians and variance measures should be available. If there are restrictions on publicly sharing data—e.g. participant privacy or use of data from a third party—those must be specified.

Reviewer #1: Yes

5. Is the manuscript presented in an intelligible fashion and written in standard English?

PLOS ONE does not copyedit accepted manuscripts, so the language in submitted articles must be clear, correct, and unambiguous. Any typographical or grammatical errors should be corrected at revision, so please note any specific errors here.

Reviewer #1: Yes

6. Review Comments to the Author

Please use the space provided to explain your answers to the questions above. You may also include additional comments for the author, including concerns about dual publication, research ethics, or publication ethics. (Please upload your review as an attachment if it exceeds 20,000 characters)

Reviewer #1: Thank you for submitting the revised manuscript. I am satisfied with the authors response to the reviewers’ comments and believe that the manuscript should be accepted. I would however like to suggest a couple of minor changes for the authors to consider.

1. In lines 304-305 you state “Testing of urines from children with lower respiratory tract infection, none of whom had pneumococcal pneumonia, was performed to determine the specificity of urine antigen testing...”
A number of publications have highlighted that the diagnosis of pneumonia caused by pneumococci can be insensitive when based solely on culture. In fact, this is a reason in favour of the use of urine antigen diagnostic kits such as the BinaxNOW and ImmuView. For this reason, I believe that it is incorrect to state that these children did not have pneumococcal pneumonia and instead suggest that you re-word this sentence to note that the children were deemed not to have pneumococcal pneumonia based on the absence of any culture.

**Thank you. The sentence has been rewritten to say (changed text is underlined): “Testing of urines from children with lower respiratory tract infection, none of whom had pneumococcal pneumonia based on the absence of a positive culture result for S. pneumoniae, was performed to determine the specificity of urine antigen testing since nasopharyngeal pneumococcal colonization is known to lead to falsely-positive urine antigen assays.”**

2. Lines 327-328 – “For the three BinaxNOW-ImmuView discordant result UPenn L pneumophila results for urines from patients with Legionnaires' disease…” – perhaps consider removing the first "result" from this sentence for clarification.

**Thank you. This extraneous word “result” was removed as suggested. The sentence now reads; “For the three BinaxNOW-ImmuView discordant UPenn *L pneumophila* results for urines from patients with Legionnaires' disease, only one discrepancy was resolved after boiling treatment.”**
